# Microstructure Features and High Temperature Oxidation Resistance of In-Situ TiN-Ti Composite Coatings by Plasma Transferred Arc Welding

**DOI:** 10.3390/ma13214882

**Published:** 2020-10-30

**Authors:** Mengying Chen, Baoming Shi, Shiming Huang, Xuefei Qin, Yuan Feng, Zhaorui Yu

**Affiliations:** School of Materials Science and Engineering, Dalian Jiaotong University, Dalian 116028, China; cmy05091@163.com (M.C.); S1455291509@163.com (B.S.); qxf1259017853@163.com (X.Q.); fy15941016132@163.com (Y.F.); djtuyzr@163.com (Z.Y.)

**Keywords:** high temperature oxidation, composite coatings, microstructure, in-situ

## Abstract

In order to improve the high temperature oxidation resistance of Ti6Al4V alloy, the in-situ TiN-Ti composite coatings were prepared with Ti-Cr-Ni-Nb powders by plasma transferred arc welding. Nitrogen gas was used as the transport gas and provided N source for the formation reaction of TiN. Microstructure features and high temperature oxidation behaviors of the composite coatings were studied. The phases in the composite coatings were TiN, Ti, CrN, and NiTi. It was clearly observed that in-situ TiN particles were evenly distributed in the Ti matrix. A little Nb atom dissolved in TiN particles, and others dissolved in the Ti matrix. By comparing the curve of Ti6Al4V alloy to that of the composite coatings, the oxidation mass gain of the composite coatings was comparatively less. The oxidation film of the composite coatings was smooth and compact, and no crack was visibly observed. Based on the results of the high temperature tests, the composite coatings exhibited superior high temperature oxidation resistance than Ti6Al4V alloy both at 650 °C and at 850 °C.

## 1. Introduction

Titanium alloys have been used extensively across various industries due to the advantages, such as the low density, the high specific strength, the good corrosion resistance, and the excellent biocompatibility [1,2]. Some scholars around the world have realized the importance of titanium alloys and focused on the research and development of them [3,4]. At present, the application of titanium alloys has been involved in the aviation, shipbuilding, automobile, marina, and medical industries. Generally, the usability of titanium alloys has a strict requirement for the surface integrity of the components and parts [5]. The retting wear, frictional wear, and corrosive pitting might happen frequently on the surface of titanium alloys resulting from being easily oxidized at high temperatures [6,7,8]. Therefore, researchers have begun to pay relatively high levels of attention to the surface treatment technology of titanium alloys. The surface treatment technology, which is applicable to titanium alloys, includes hot dipping [9,10], vapor deposition [11], thermal spray [12,13], and laser cladding [14,15,16,17,18]. Research increasingly shows that these technologies can improve the high temperature oxidation resistance, corrosion resistance, wear resistance, and biological activity of titanium alloys [19,20,21].

Traditional coatings consisting of a single component are hard-pressed to meet the increasingly demanding of the practical application of materials. For instance, although cermet has good oxidation resistance and thermal stability, they are hard to prepare on metallic materials due to the poor wettability. The metal matrix composite coatings have a high specific strength and an excellent heat resistance compared with the conventional metal material coatings. In addition, they also have a high toughness and a high impact resistance in contrast with the ceramic material coatings. The cooperation between each component in the composite coatings can make heavy use of the advantages and make up for the disadvantages. As a consequence of this, the metal matrix composite coatings show a huge superiority compared with the coatings with a single component. Pougoum et al. [22] comparatively studied the wear behavior of ex-situ and in-situ TiN-TiB_2_ reinforced Fe-based coatings prepared by HOVF. The in-situ composite coatings had fine and uniform reinforced ceramic particles compared with the ex-situ composite coatings, and hence the in-situ coatings exhibited higher microhardness and wear resistance. Furthermore, there existed fractured ceramic particles with shape edge due to the poor interface between the particles and the matrix. Liu et al [23] had successfully prepared TiN/Ti_3_Al coatings on Ti6Al4V alloy via laser cladding technology. The microhardness and high temperature oxidation resistance of the coatings were significantly improved by the reinforced phase TiN. However, the pores and cracks in the coatings would cause the fracture of the oxidation film under high temperature because the internal stress in these positions was very high. Li et al. [24] utilized the first principles method in order for the stability and mechanical properties of (Ti, Nb) C particles in the metal matrix coatings. The results indicated that the lattice constants and mismatching between (Ti, Nb) C particles and Fe matrix were changed with different powders resulting in the increase of the brittleness of the reinforced particle which would be a harmful effect on the properties of the composite coatings. The composite coatings are composed of the reinforced particles and the matrix, and the interface has a great influence on the properties of the composite coatings. Consequently, an appropriate interface between the reinforced particles and the matrix is the premise of the guarantee of the properties of the composite coatings.

However, the reports on the high temperature oxidation behaviors of TiN reinforced coatings prepared by plasma transferred arc welding are very few. In this paper, in-situ TiN-Ti composite coatings were fabricated on Ti6Al4V with Ti-Cr-Ni-Nb powders by plasma transferred arc welding. In order to avoid the produce of harmful particles due to the addition of other nitride powders, nitrogen gas was used as the transport gas and provided an N source for the formation reaction of TiN. This can not only reduce the cost but also simplify the process. The microstructure characterization of the composite coating was investigated. The high temperature oxidation tests at 650 °C and at 850 °C were carried out to study the high temperature oxidation behaviors of the composite coatings, and the oxidation mechanisms were explained.

## 2. Materials and Methods

### 2.1. Materials

**Figure 1:**
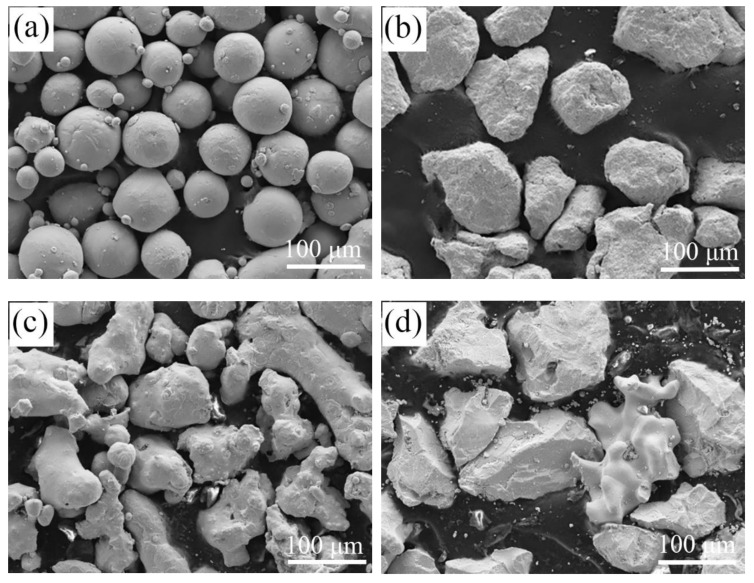
SEM morphologies of the powders: (**a**) Ti; (**b**) Cr; (**c**) Ni; and (**d**) Nb.

A commercial Ti6Al4V alloy (Shenyang nonferrous metal Ltd. company, Shenyang, China), whose chemical composition was Ti-6.5Al-4.26V-0.1C in a weight ratio, was selected as the substrate. The substrate was divided into several plates with the dimensions of 100 mm × 80 mm × 10 mm, and, after that, the plates were cleaned in an ultrasonic cleansing machine to remove the oil and heated at the temperature of 150 °C for 1 h in a resistance furnace. The raw materials were a mixture of pure Ti powders, pure Cr powders, pure Ni powders, and pure Nb powers (All the powders were fabricated by Jiangsu Vilory Ltd., 99.98 wt.%). The powders with the weight ratio of 7:1:1:1 (Ti:Cr:Ni:Nb) were adequately mixed and then kept in a resistance furnace at 100 °C for 2 h prior to the preparing of the coatings. Figure 1 depicts the SEM morphologies of the powders. As shown, pure Ti powders (D50 89.85 μm) are spherical atomized powders with a range of sizes from 50 μm to 120 μm. Pure Cr (D50 97.82 μm), pure Ni (D50 110.53 μm), and pure Nb (D50 105.18 μm) powders are crushed block-shaped.

### 2.2. Preparing of In-Situ TiN-Ti Composite Coatings

**Figure 2:**
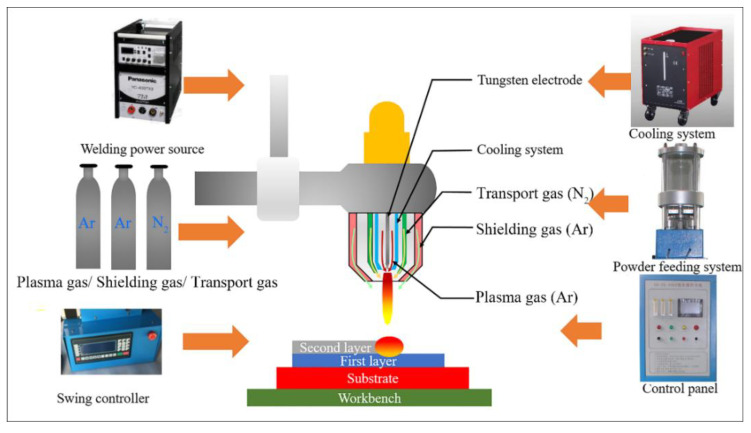
Schematic diagram of plasma transferred arc welding.

In-situ TiN-Ti composite coatings were fabricated by using a plasma transferred arc welding machine (Shanghai Benxi Ltd. Company, Shanghai, China). The schematic diagram of plasma transferred arc welding is shown in Figure 2. Nitrogen gas (99.99% purity) was used as the transport gas, and argon gas (99.99% purity) was used as the plasma gas and the shielding gas. The welding parameters were optimized in order to obtain the composite coatings with good appearance and with no defects, such as surface cracks and pores. The optimized parameters were described below: welding current 64A, welding speed 1.2 mm/s, powder feeding rate 8.8 g/min, plasma gas flow 4.5 L/min, transport gas flow 5.0 L/min, and shielding gas flow 6.0 L/min. The specimens after plasma transferred arc welding were cooled slowly in the air.

### 2.3. Phase and Microstructure Analysis

The surface of the specimens with coatings was cut into a flat surface by a wire cutting machine, and then grinded successively with 180# sandpapers, 600# sandpapers, and 1000# sandpapers. The phase composition of in-situ TiN-Ti composite coatings were analyzed by a X-ray diffraction analyzer (XRD, Empyrean, Almelo, the Netherlands) with Cu-Kα radiation at 40 mV and 40 mA. The cross section of the specimens was grinded, polished, and eroded with a mixture of 30 mL HNO_3_, 10 mL H_2_O, and 5 drops of HF for 15 s. Scanning electron microscope (SEM, SUPRA55, ZEISS, Jena, Germany) was used to analyze the microstructure of composite coatings. The interface between the reinforced particles and the matrix was further investigated by a transmission electron microscope (TEM, JEM-2100F, Japan Electronics Co. LTD, Tokyo, Japan).

### 2.4. High Temperature Oxidation Tests

High temperature oxidation tests were conducted by a KSY-16-15 box-type resistance furnace (Shanghai Shiyan Ltd. Company, Shanghai, China). The samples with the dimensions of 12 mm × 10 mm × 4 mm were used for oxidation tests. The coatings were tested without the substrate. Before the tests, the samples were grounded to 1000# sandpapers and polished with the diamond polishing plate. The roughness is in the range of 100 nm to 200 nm. Then, the superficial area and the initial weight of the samples were recorded. The isothermal oxidation tests were carried out at 650 °C and at 850 °C, respectively. When the temperature reached the target temperature, the samples together with porcelain vessels were put in the resistance furnace. A set of samples were taken out after 1 h, 5 h, 12 h, 24 h, 36 h, 48 h, 60 h, 72 h, 84 h, 96 h, 108 h, and 120 h, respectively. The oxidized samples were weighed again. According to the mass variation, the mass gain curves of the samples were obtained. XRD and SEM with EDS was used to analyze the phase, the morphologies, and the chemical composition of the oxidation film.

## 3. Results

### 3.1. XRD Results

**Figure 3:**
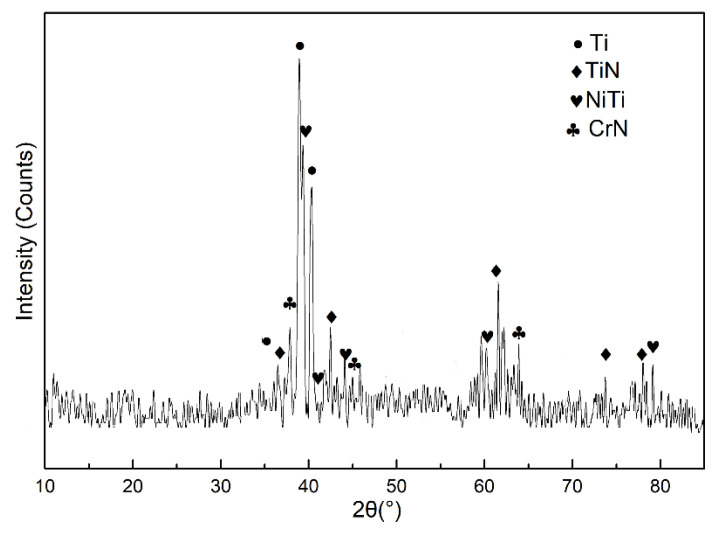
XRD pattern of the composite coatings.

The XRD pattern of in-situ TiN-Ti composite coatings is given in Figure 3. It is observed that the main phases in the composite coatings are TiN, Ti, CrN, and NiTi. There was no TiN, CrN, and NiTi in the raw materials, and hence these phases were formed during the plasma transferred arc welding process. Compared with the standard diffraction peaks of Ti, there is an obvious deviation of angle to the left, which means that some solute atoms with a larger atomic radius are dissolved in the Ti matrix. This may be attributed to the lattice distortion resulting from the dissolving of Nb atoms in Ti matrix according to the fact that no compounds containing Nb are found in the XRD results.

### 3.2. Microstructure Features

**Figure 4:**
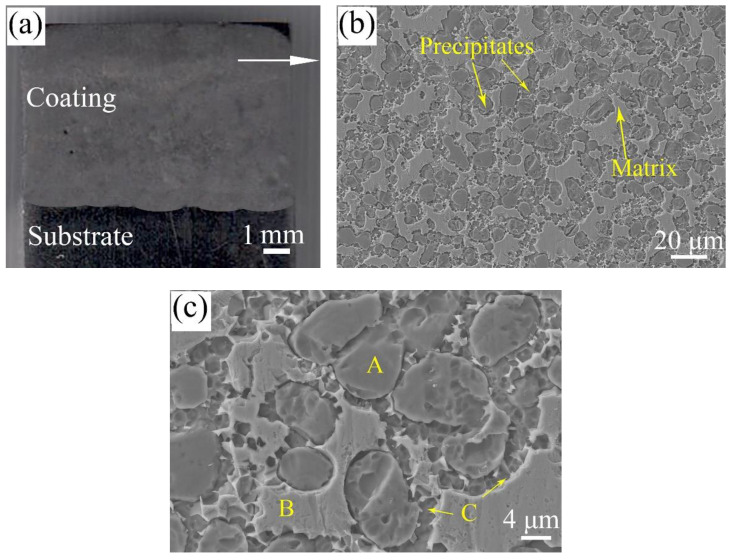
Morphologies on the cross section of the composite coatings: (**a**) macro-morphology; (**b**) the typical microstructure; and (**c**) high magnification.

Figure 4 is the morphologies of in-situ TiN-Ti composite coatings. As demonstrated in Figure 4a, the composite coating is well metallurgically bonded to the substrate with a low dilution rate. The upper microstructure of the composite coating is also given in SEM. A number of precipitates are uniformly dispersed in the grey matrix. At high magnification, it can be seen that most of the precipitates (A) are spherical or elongated. In addition, the interface between the precipitates and the matrix (B) is concave. There are many sunken particles (C) embedded in the interface between the precipitates and the matrix. Therefore, the interface is seemingly irregular and rough.

**Figure 5:**
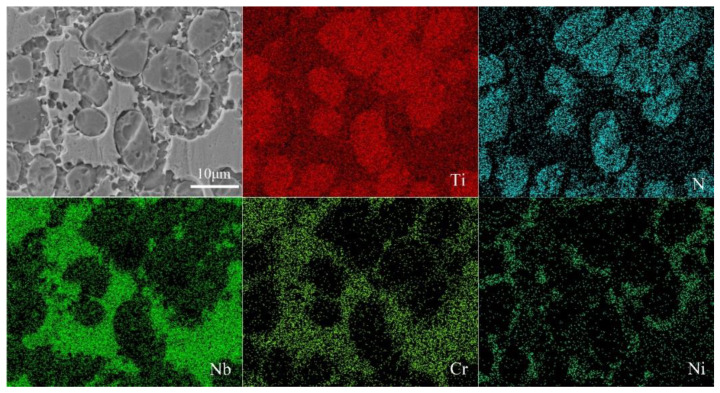
Distribution of element in the typical microstructure of the composite coatings.

EDS was utilized for the analysis of the chemical composition of the typical microstructure. Figure 5 is the distribution of element in the typical microstructure. It is clearly observed that the precipitates are rich in Ti and N elements, and Cr elements primarily spread over the matrix. Most of the Nb elements are distributed in the matrix, and a small amount of Nb element can be detected distributed in the precipitates. Moreover, the Ni element is concentrated in the concave interface.

**Figure 6:**
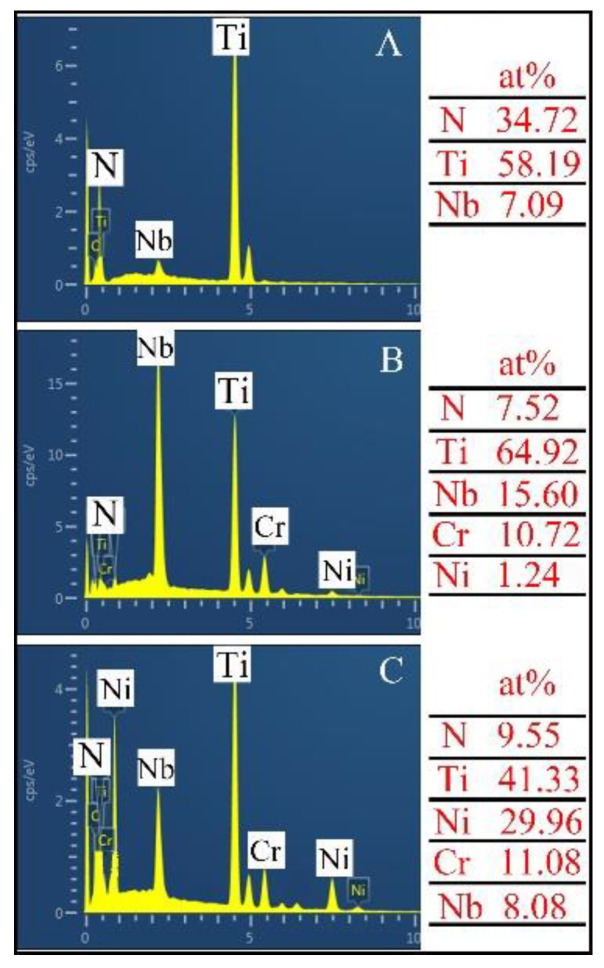
EDS results of the marked locations in Figure 4c.

Figure 6 is the EDS results of the marked locations in Figure 4. The EDS results demonstrate that the precipitates are composed of 58.19 at.% Ti-7.09 at.% Nb-34.72 at.% N. According to the XRD results and the proportion of each element, it can be inferred that the precipitates (A) are TiN particles. It is worth mentioning that TiN particles were not added into the raw materials beforehand. In other words, TiN particles were in-situ formed by dint of the reaction between Ti powders and N_2_ under the effect of the plasma arc. During plasma transferred arc welding, Ti powders in a molten state were transported into the molten pool by nitrogen gas. Thus, Ti powders were fully exposed to nitrogen gas, and the in-situ reaction tended to easily happen. Moreover, in-situ TiN particles with a size in the range of 5–18μm evenly distribute in the matrix. The grey matrix (B) is identified as Ti matrix due to the high atomic mass percentages of Ti (64.92 at.%). Obviously, the content of Nb in Ti matrix is comparatively high. This is because a small number of Nb powders are consumed and enter into in-situ TiN particles, and other Nb atoms dissolve in the Ti matrix. Certainly, this is also in accordance with the XRD results. As depicted, the sunken particles (C) are composed of 41.33 at.% Ti-29.96 at.% Ni-9.55 at.%. N-11.08 at.% Cr-8.08 at.% Nb. It can be inferred that the sunken particles are CrN and NiTi [25]. However, it is hard to distinguish them in SEM. Hence, TEM was utilized for the further study on the microstructure of the composite coatings.

**Figure 7:**
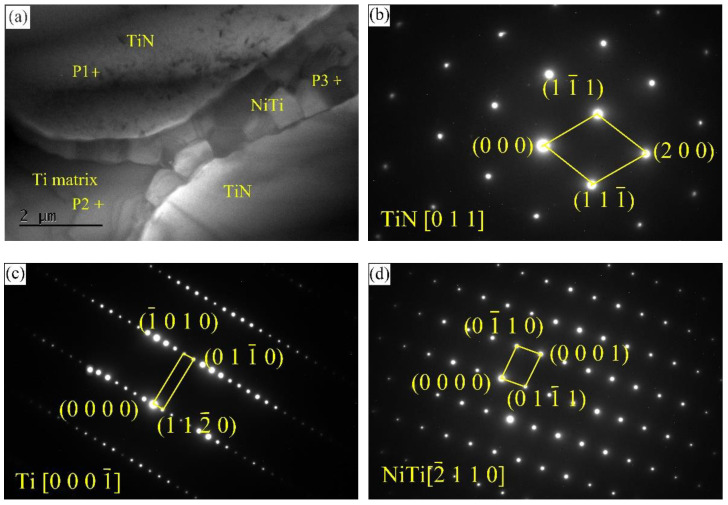
TEM image and SAED patterns of the composite coating: (**a**) TEM image; (**b**–**d**) SAED patterns.

The TEM image and the selected area electron diffraction (SAED) patterns of the composite coating are shown in Figure 7. The EDS results of the marked locations are given in Table 1. According to the EDS results and the SAED patterns in Figure 7b,c, in-situ TiN particles with face-centered cubic structure are identified, and the matrix is regarded as a Ti matrix with a hexagonal close-packed structure. As demonstrated in Figure 7a, several blocky particles with uniform size (about 1 μm), which have a similar appearance to the sunken particles in Figure 4c, are observed in the interface. The SAED pattern along the zone axis of [-2 1 1 0] demonstrates that the blocky particles are NiTi particles. NiTi particles are evenly embedded in the interface, and the pinning effect of NiTi particles can be conducive to the enhancing of the performance of the composite coatings.

### 3.3. High Temperature Oxidation

**Figure 8:**
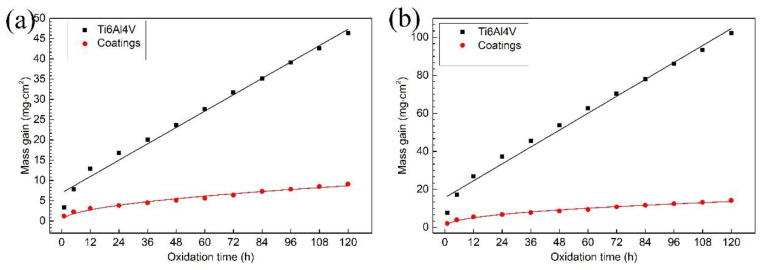
Oxidation mass gain curves of the samples: (**a**) at 650 °C; (**b**) at 850 °C.

The oxidation kinetics law is influenced by oxidation temperature and oxidation time. Thus, generally speaking, the mathematic relation between oxidation mass gain and oxidation time is used to explain the oxidation behavior of materials. Figure 8 shows the mass gain curves of the samples in the isothermal oxidation tests. By comparing the curve of Ti6Al4V alloy to that of the composite coatings, the oxidation mass gain of the composite coatings is comparatively less. This illustrated that the oxidation film of the composite coatings could prevent them from being oxidized further. From Figure 8a, it is clearly evident that the oxidation kinetics law of Ti6Al4V alloy is linear, which demonstrates that this oxidation film has poor oxidation resistance. The oxidation mass gain of the composite coatings is relatively high at an earlier stage, and then tends to be stable gradually. A parabola relationship between oxidation mass gain and oxidation time is presented. Therefore, the oxidation film on the surface of the composite coatings is protective and could lower the oxidation rate. As demonstrated in Figure 8b, the oxidation kinetics law at 850 °C is similar to that at 650 °C. It can been seen that the oxidation mass gain of Ti6Al4V alloy is larger, and the oxidation rate is also higher. The mass gain of the composite coatings is 14.2 mg/cm^2^ after isothermal oxidation tests for 120 h, which decreases by 86.1% compared with that of the substrate (102.1 mg/cm^2^). Though the mass gain of the composite coatings at 850 °C is larger than that at 650 °C, on the whole, the relationship between oxidation mass gain and oxidation time still remains in the shape of a parabola. As a rule, the higher the temperature is, the more violent the atomic motion is. This is why the mass gain increased. 

**Figure 9:**
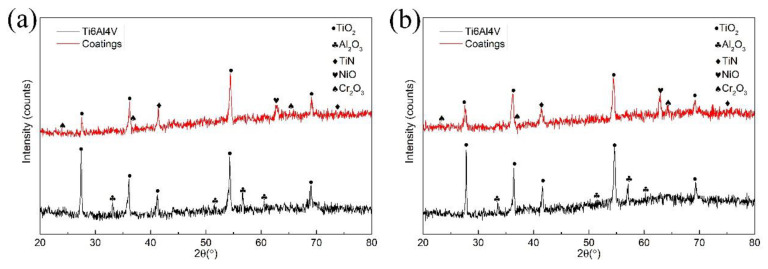
XRD patterns of the surface oxidation film of the samples: (**a**) at 650 °C; (**b**) at 850 °C.

Figure 9 is the XRD patterns of the surface oxidation film of the samples after isothermal oxidation tests for 120 h. After isothermal oxidation tests, the phases of the oxidation film of Ti6Al4V alloy are TiO_2_ and Al_2_O_3_. The peak intensities of TiO_2_ are apparently higher than that of Al_2_O_3_, which demonstrates that the oxidation of Ti6Al4V alloy is largely composed of TiO_2_ oxide. Due to the lower Gibbs free energy of Al_2_O_3_, Al_2_O_3_ exists in the oxidation film [26]. In the XRD patterns of the composite coatings after isothermal oxidation tests, the diffraction peaks of TiO_2_, TiN, Cr_2_O_3_, and NiO. Compared with the diffraction peaks of TiN before isothermal oxidation tests, the intensities reduce visibly due to the coverage of the oxidation film and the consumption of the oxidation reaction. The appearance of Cr_2_O_3_ and NiO illustrates that CrN and NiTi have reacted with oxygen during the isothermal oxidation tests. In addition, there is no obvious difference between the XRD patterns of the composite coatings at 650 °C and 850 °C.

**Figure 10:**
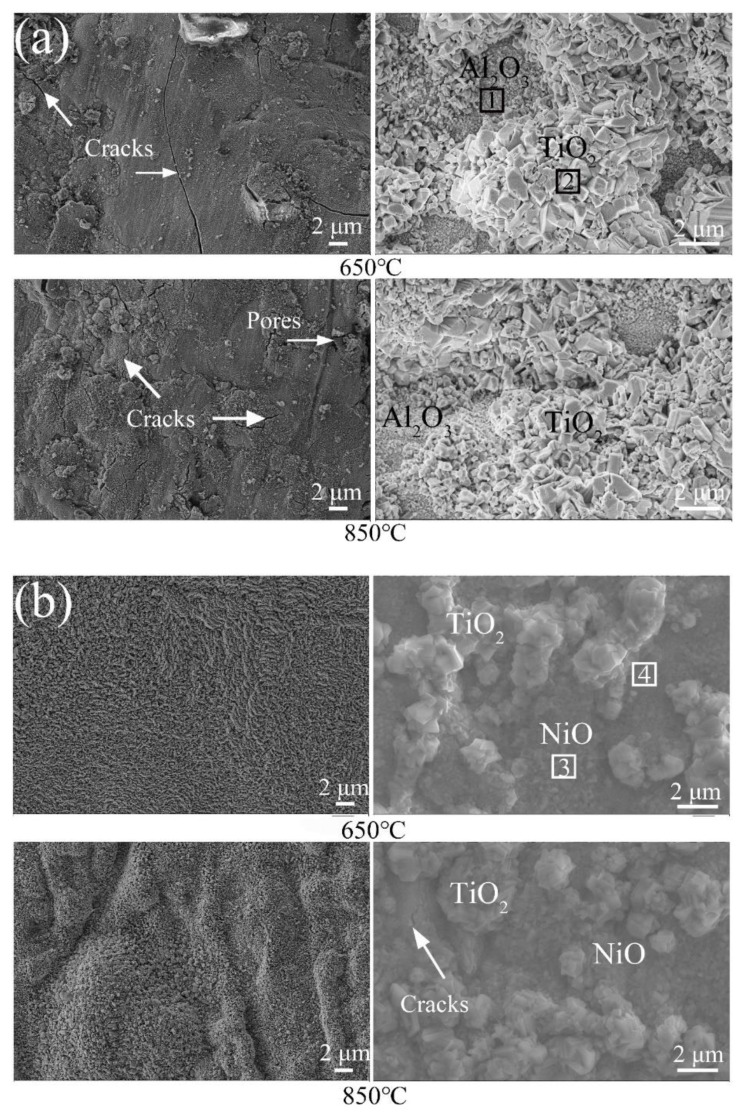
Surface oxidation film of the samples after isothermal oxidation tests for 120h: (**a**) Ti6Al4V alloy; (**b**) the composite coatings.

**Figure 11:**
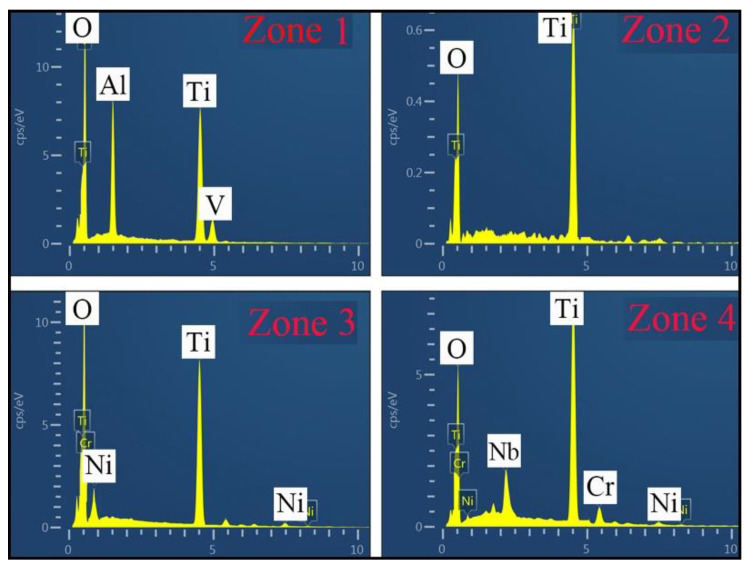
EDS results of the marked locations in Figure 10.

Figure 10 shows the surface oxidation film of the samples after isothermal oxidation tests for 120 h. Figure 11 gives the EDS results of the marker locations in Figure 10. From Figure 10a, the surface oxidation film of Ti6Al4V alloy is composed of a large number of TiO_2_ and a small number of Al_2_O_3_ [26,27]. Cracks and pores were observed. TiO_2_ with greater brittleness will be liable to cracking and fall out. Moreover, the looser structure will provide access to oxygen and accelerate its diffusion [28]. In addition, TiO_2_ oxide has a relatively low dissociation pressure at a temperature of 2000 K. As a result, Ti6Al4V alloy will still continue to react with oxygen even though its surface is covered with TiO_2_ oxidation film. By comparing the surface of Ti6Al4V alloy at 650 °C and at 850 °C, there are more cracks in the oxidation film at 850 °C. From Figure 10b, the surface oxidation film of the composite coatings is mainly composed of a mixture of oxides, such as TiO_2_ and NiO [29]. In zone 4, the chemical compositions are composed of 27.35 at.% Ti-62.18 at.% O-7.85 at.%. Nb-1.75 at.% Cr-0.87 at.% Ni. Due to the dissolving of Nb in Ti, Ti_x_Nb_1-x_O_2_ will be formed by the means of the replacing of Ti^4+^ ions in TiO_2_ by Nb^5+^ ions [30,31]. Compared with the oxidation film of Ti6Al4V alloy, that of the composite coatings is more compact. This oxidation film formed a protective layer with the function of hindering the transmission of oxidation, and hence the oxidation process was weakened. However, small cracks are observed in the composite coatings at 850 °C. Consequently, this can be used to explain why the mass grain at 850 °C is larger than that at 650 °C.

**Figure 12:**
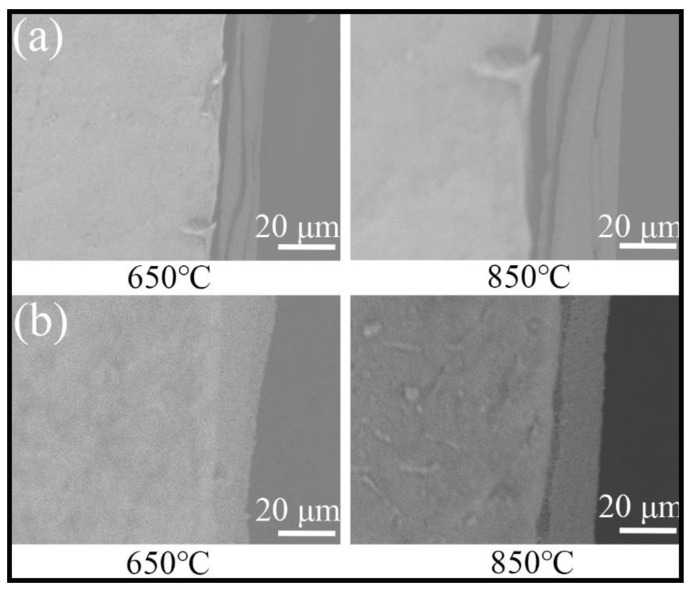
Cross-sectional SEM of the samples after isothermal oxidation tests for 120h: (**a**) Ti6Al4V alloy; (**b**) the composite coatings.

Figure 12 is the cross-sectional SEM of the samples after isothermal oxidation tests for 120 h. As shown, some long and deep cracks appear in the oxidation film of Ti6Al4V alloy. Furthermore, the oxidation film is not bonded to the unoxidized substrate tightly. The formation and peeling of the oxidation film will repeat again and again. In this case, it is easy for oxygen to diffuse into the interior so that the oxidation process cannot be suppressed. By contrast, the compact and stable oxidation film of the composite coatings acted as an oxygen barrier and decreased the content of dissolved oxygen. The close integration between the oxidation film and the composite coatings can ensure that the oxidation film will not peel easily.

At the earlier stage of oxidation, in-situ TiN particles and Ti matrix in the composite coatings, which are exposed to the air directly, are oxidized rapidly, and then TiO_2_ crystal nucleus are formed. Meanwhile, CrN and NiTi particles with small sizes offer the source of Cr and Ni for the oxidation reactions of Cr_2_O_3_ and NiO. The oxidation process at this time takes place under the condition that the composite coatings are in immediate contact with oxygen because the oxidation film is thin. As a result, the oxidation mass gain is larger at the earlier stage of oxidation. As the oxidation process continues, the thickness of the oxidation film increases. The oxidation reactions depend on the diffusion of oxygen in the oxidation film towards the composite coatings. Thus, the oxidation rate becomes slow. Due to a mass of in-situ formed TiN particles, the newly formed TiO_2_ particles flock together resulting in the produce of a lamellar and compact oxidation film [32,33,34]. Moreover, Cr_2_O_3_ and NiO oxides generate and grow alternately in the oxidation process so that the oxidation film is more compact and bonded to the substrate tightly [35]. Consequently, the diffusion rate of oxygen towards the substrate is lower, thereby weakening the oxidation reactions. Furthermore, Nb atoms that dissolve into TiN particles and Ti matrix have an effect of solution strengthening and second phase strengthening [36]. The density of oxygen vacancy will decline in this manner. Accordingly, the mobility ratio and diffusion of oxygen are reduced. In conclusion, the composite coatings have superior high temperature oxidation resistance both at 650 °C and at 850 °C.

## 4. Conclusions

(1) In-situ synthesized TiN-Ti composite coatings were obtained by plasma transferred arc welding. The in-situ reactions of reinforced particles could proceed smoothly because the powders were fully exposed to nitrogen gas, which was used as the transport gas.
(2) In-situ TiN particles were uniformly distributed in a Ti matrix. A small number of Nb powders entered into in-situ TiN particles, and other Nb atoms dissolved in the Ti matrix.
(3) Due to the compact and smooth oxidation film, the composite coatings exhibited superior high temperature oxidation resistance than Ti6Al4V alloy both at 650 °C and at 850 °C.

## Figures and Tables

**Table 1:** EDS results of the marked locations in Figure 7.

Marked Locations	Ti (at.%)	Cr (at.%)	Ni (at.%)	Nb (at.%)	N (at.%)
P1	55.14	-	-	5.65	39.21
P2	63.19	12.47	1.03	18.64	4.67
P3	51.91	2.16	43.69	2.24	-
